# Development of simulated arthroscopic skills: A randomized trial of virtual-reality training of 21 orthopedic surgeons

**DOI:** 10.3109/17453674.2011.552776

**Published:** 2011-02-10

**Authors:** Christine Andersen, Trine N Winding, Martin S Vesterby

**Affiliations:** ^1^Department of Orthopedics; ^2^Orthopedic Skills Laboratory, Silkeborg Regional Hospital, Silkeborg, Denmark

## Abstract

**Background and purpose:**

Previous studies have shown that there is a correlation between arthroscopic experience and performance on a virtual-reality (VR) unit. We analyzed the development inexperienced surgeons went through during VR training of shoulder arthroscopy.

**Methods:**

14 inexperienced surgeons from Silkeborg Regional Hospital were randomized into an intervention group and a control group. 7 experienced surgeons constituted another control group. All were tested twice on insightMIST—an advanced arthroscopic VR trainer—within a period of 6–15 days. The intervention group also received a 5-hour training program on the VR unit.

**Results:**

The average time for the arthroscopy in the intervention group was reduced from 720 (SD 239) seconds to 223 (SD 114) seconds (p = 0.03 compared to the inexperienced control group). Distance travelled by the camera was reduced from 367 (SD 151) cm to 84 (SD 44) cm in the intervention group (p = 0.02 compared to the inexperienced control group). Depth of collisions was also significantly reduced, whereas distance travelled by the probe and number of collisions were improved in the intervention group, although not statistically significantly.

**Interpretation:**

VR training is a possible way for young and inexperienced surgeons to achieve basic navigation skills necessary to perform arthroscopic surgery. Further studies regarding the transferability of the skills acquired on the VR unit to the operating theater are desirable.

Previous studies have shown that there is a correlation between arthroscopic experience and performance on a virtual reality (VR) unit, which is an advanced arthroscopic training simulator that allows users to learn and improve in minimally invasive surgical techniques. Both single-point estimates and longitudinal studies have indicated that increased real-time arthroscopic experience correlates with performance on a VR unit (Bliss et al. 2005, McCarthy et al. 2006, Ceponis et al. 2007, Gomoll et al. 2008, Howells et al. 2008b). There have been no studies, however, showing the development that inexperienced orthopedic surgeons go through when receiving training on a VR unit, and comparing the skills they acquire to those of experienced surgeons who regularly deal with arthroscopic surgery—or to a complementary control group of inexperienced surgeons not receiving any training.

The number of arthroscopic surgical procedures is increasing. In the Danish healthcare system, where there is a high demand for productivity, it can be hard to find the time to educate the new generation of orthopedic specialists. The educational environment is not optimal for the inexperienced surgeon who is aware of the time pressure. Furthermore, patient safety must be considered. Thus, it is critical to consider alternative methods of raising the standard of education and for this purpose VR training has proven beneficial in other surgical fields, such as gynecology and laparoscopy (Larsen et al. 2006, Gurusamy et al. 2009, Kossi and Luostarinen 2009, Larsen et al. 2009). Other studies have shown that experienced surgeons have markedly better performance than inexperienced doctors on a VR unit, indicating that the skills acquired in the operating theater are transferable to the VR unit and visa versa (Pedowitz et al. 2002, Srivastava et al. 2004, Gomoll et al. 2007). We assessed the effect of training on a VR unit for a group of doctors with no arthroscopic experience. Our hypothesis was that after undergoing a training program on a VR unit, inexperienced doctors would improve compared to doctors of similar experience who did not undergo the program, and perform at the same level or better than experienced surgeons—as measured by performance on the VR unit.

## Methods

21 doctors working at Silkeborg Regional Hospital in May and June 2008 were included in the study. The reason for choosing 21 was mainly based on practical circumstances such as numbers of newly started interns in the department. Before enrollment, all doctors filled in a questionnaire regarding their arthroscopic experience and were divided into the following groups. Group 1—the experienced control group—consisted of 7 experienced arthroscopic surgeons who did at least 1 independent arthroscopic procedure a week. Group 2—the intervention group—consisted of 7 interns from the orthopedics department with no arthroscopic experience. Group 3—the inexperienced control group—consisted of 7 interns from the same hospital with no arthroscopic experience. The interns were randomized into either the control or the intervention group by drawing lots. The questionnaire also included questions regarding the amount of time spend on computer games by the subjects on a daily basis and showed no difference between the 3 groups.

### Data collection

**Figure 1.**
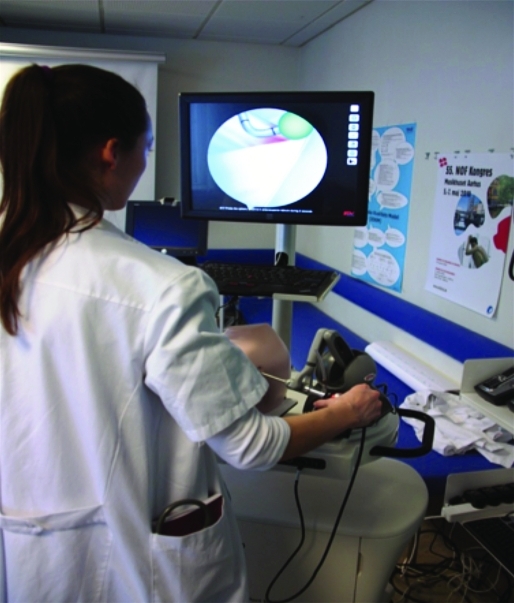
InsightMIST.

All the subjects went through a standard test in shoulder arthroscopy on insightMIST—an advanced arthroscopic virtual-reality trainer manufactured by GMV (Tres Cantos, Madrid, Spain). The set-up is illustrated in Figure 1.

The unit was chosen after testing 2 different VR simulators, and the GMV VR trainer provided the more realistic VR environment both regarding the forced feedback and the anatomical visualization. Furthermore, GMV showed great interest in continued development and improvement of the system. The unit consisted of a high-performance computer, a 19-inch touch monitor, 2 haptic devices (SensAble PHANTOM; 1 simulated camera and 1 simulated probe), 1 integration platform with dynamic elements, and 1 physical (left) shoulder model.

**Figure 2.**
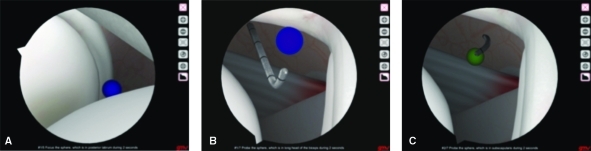
Locating the sphere (A). Palpating the sphere with the probe (B). Touching the sphere for 2 seconds (C).

The purpose of the test was to complete a shoulder arthroscopy by identifying a number of spheres in the joint placed on anatomic landmarks, centering the spheres one by one with a camera and palpating them with a probe, as illustrated in Figure 2. The sequence by which the spheres were placed changed from test to test, making it impossible to know the position of the next sphere. The test was executed as follows. The subject was alone in the training room during the test, only accompanied by the instructor. Before the test, the subject was handed standard written instructions explaining the purpose of and how to complete the test. Before starting the test, the subject had the opportunity to discuss and clarify any doubts with the instructor. The instructor was the same person throughout the experiment and was present through the entire duration of the test, but was not allowed to give any advice during the test. All the subjects underwent the test twice with a minimum of 6 days between tests. In the intervening period, the control groups were not allowed to use the VR unit at all. For practical reasons, the upper time limit to complete the test was set at exactly 15 min.

**Figure 3.**
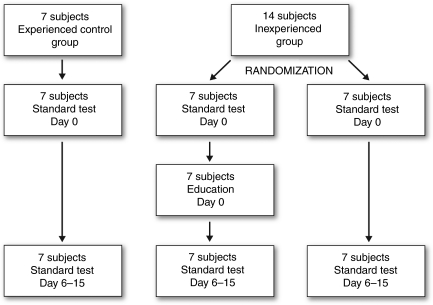
Flow chart of the experiment.

The intervention group started their training day by completing the standard test in the same way as the subjects in the control groups. After completing the standard test, they did not repeat that particular test again until they were retested approximately 1 week later, as illustrated in Figure 3. Instead, they went through a 5-hour training program on the VR unit, starting out by receiving advice in basic navigation skills by a surgeon of more experience than themselves (although not an specialist in orthpedics). After that, they went systematically through the tests available on the VR unit. An instructor was present throughout the day and was available for guidance. After completing the 5-hour program, the subjects were not allowed to use the VR unit before repeating the standard test on the day of follow-up.

All the subjects were evaluated on 5 different parameters: time to complete the exercise, number of collisions with surrounding tissue, maximum depth of collision with surrounding tissue, and paths travelled with both camera and probe. All the data were computed automatically and no manual registration was involved.

### Statistics

The data were normally distributed and were analyzed with a t-test using Stata statistical software version 9.0. A p-value of < 0.05 was considered to be statistically significant.

## Results (Figure 4)

The 21 subjects all completed the standard test twice at a mean interval of 10 days. We intended to keep this interval at around 1 week since this was estimated to be an appropriate and realistic length of time for future interns between the training session on the VR unit and doing supervised but real-time arthroscopic surgery in the theater. The intervention group and the inexperienced control group were judged in a similar way by their performance in test 1, based on the 5 different parameters (Table 1).

**Figure 4.**
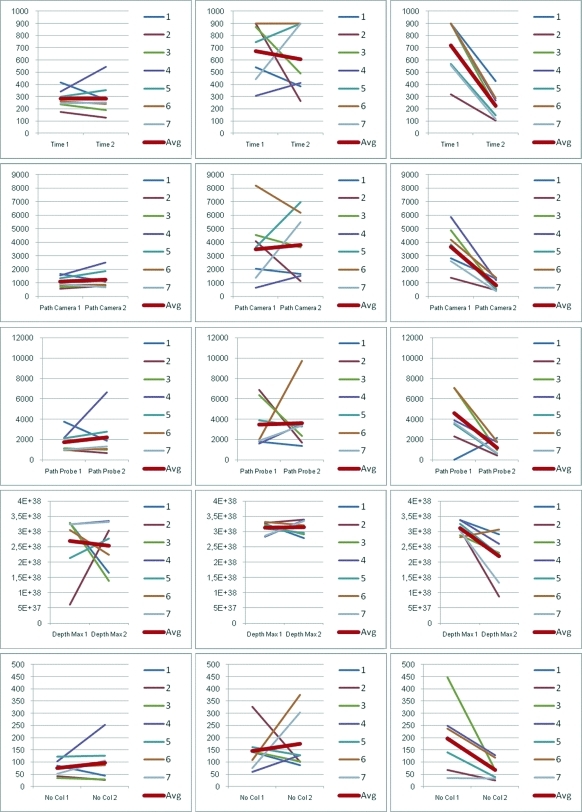
Individual development from the first to the second test concerning the 5 different parameters, for all 3 groups. Group 1: experienced control group; group 2: inexperienced control group; group 3: intervention group

**Table 1.** Comparing group 2 (inexperienced control) and group 3 (intervention) in test 1

	Difference between the groups Group 3 – Group 2	P-value comparing the groups
Time	47 s	0.7
Distance camera	162 mm	0.9
Distance probe	1099 mm	0.4
Depth of collisions	–0.02 kN	0.9
Number of collisions	52	0.4

The results for the intervention group were all characterized by a vast improvement from the first to the second test, measured using all 5 parameters. The experienced control group had only minor variations between the 2 tests. The inexperienced control group was characterized by what appeared to be random results, with large inter- and intrapersonal variation.

The intervention group went from an average of 720 seconds in the first test to 223 seconds in the second test, an average improvement of 497 seconds. The average improvement for the inexperienced control group was 65 seconds, whereas the experienced control group neither increased nor decreased their average time consumption, since the difference was only 1 second. The intervention group improved more than the inexperienced control group (p = 0.03). After going through the training program, the intervention group completed the exercise faster that the experienced control group, but this difference was not statistically significant (p = 0.4).

The same tendency was seen when analyzing distance travelled by the camera. The intervention group had an average improvement of 283 cm for the camera whereas both of the control groups increased this distance from the first to the second test. The intervention group showed the worst result in the first test, but the best in the second test. The improvement made by the intervention group compared to the inexperienced control group was statistically significant (p = 0.02).

The distance travelled by the probe showed the same tendency as for distance travelled by camera, although the improvement made by the intervention group was not statistically significant compared to the inexperienced control group. The intervention group provided a better result in the second test than the experienced control group on distance travelled by probe and camera, although the numbers could not be shown to be statistically significantly different.

Analysis of the potential damage caused to the joint by considering the number of times either camera or probe collided with surrounding tissue, and the force by which the instruments collided, also showed a vast improvement in the intervention group—which went from causing the highest number of collisions in the first test to the lowest in the second test. The average improvement was 129 less collisions for the intervention groups, whereas the number of collisions increased in both of the control groups. The improvement was not statistically significant (p = 0.07) when comparing the intervention group with the inexperienced control group. Regarding the depth of collisions, the intervention group improved more than the inexperienced control group (p = 0.02) (Table 2; see supplementary data).

## Discussion

After completing a 5-hour training program on a VR unit, the 7 doctors who had never performed independent arthroscopic procedures showed marked improvement in their skills, placing them on the same level or making them even better than experienced surgeons (also measured on performance on the VR unit).

Our study was based on a small number of subjects, thus providing some statistical uncertainty since minor variations in the subjects' performance had a relatively large effect on the outcome. This was seen, for example, with one of the subjects in the experienced control group who provided a good result measured on all the parameters in the first test, whereas the performance in the second test was markedly worse—and worse than all the other subjects in that group. The second test probably did not reflect the subject's skills, but might have been the result of a bad day, and given the small size of the groups the result provided by that particular person affected the average results rather much.

We have found no reviews on virtual-reality training in arthroscopy and related topics. In the field of laparoscopic surgical training, we found 1 review published by the Cochrane Collaboration in January 2009. Gurusamy et al. (2009) included 23 studies (24 references) that were all randomized clinical trials. The conclusion was that “Virtual reality training can supplement standard laparoscopic surgical training of apprenticeship and is at least as effective as video trainer training in supplementing standard laparoscopic training. Further research of better methodological quality and with more patient-relevant outcomes is needed.”

The number of studies focusing on individual effects of VR training and the ability to transfer skills gained on a VR unit to the operating room is rising. Hariri et al. (2004) found that using a surgical simulator was as least as effective as textbook images for learning anatomy and, more importantly, the students: “rated the simulator higher as an effective learning tool than the textbook group rated the textbook”. McCarthy et al. (2006) showed: “significant improvements in: task completion time, shorter arthroscopic path lengths, shorter probe path lengths, and fewer arthroscopic tip contacts”. 2 control laboratory studies showed a correlation between surgical experience and an objective assessment of arthroscopic skills evaluated with a VR simulator (Gomoll et al. 2007, Howells et al. 2008a, b). Howells et al. (2008b) showed in a randomized clinical trial that the orthopedic surgical trainees who had undergone a period of laboratory-based arthroscopic simulator training demonstrated improved technical performance in the operating theater compared to an untrained group. A follow-up study showed that the skills gained in a virtual environment could still be found (in 4 of 5 parameters) when the subjects were retested after 3 years (Gomoll et al. 2008).

In spite of the small number of subjects, our study shows beneficial effects of training on a VR unit for inexperienced doctors. The improvement compared to doctors who were at the same level regarding arthroscopic skills (measured by performance on the VR unit) is striking. The fact that the intervention group outperformed the experienced group in the second test indicates that the simulator does show deviations from reality. On the other hand, the mere fact that experienced surgeons generally performed markedly better than inexperienced doctors on a VR unit indicates that the skills are somewhat transferable; some of the skills necessary to perform arthroscopic surgery such as hand-eye coordination, triangulation, and the ability to work in 3 dimensions while watching a 2-dimensional screen are skills that can be trained using a VR unit. This can lead to improved accuracy, a reduced number of errors, a reduced number of unnecessary movements, and reduced time consumption—skills that are necessary in order to perform arthroscopy in an operating theater as well as on a simulator. It would be of interest, however, to compare the performance of doctors with no VR training to doctors with VR training in an operating theater. It would also be desirable to perform similar studies on larger populations to increase the statistical certainty, and also to expand the follow-up period to investigate whether the improvements made by the intervention group would be as strong after several months or even years.

## Supplementary data

Table 2 is available at our website (www.actaorthop.org), identification number 3843.
