# Supplementary material for: Development of simulated arthroscopic skills: A randomized trial of virtual-reality training of 21 orthopedic surgeons
Source: Acta Orthop. 2011 Feb 10;82(1):90–5. doi: 10.3109/17453674.2011.552776 (PMC3230003; doi:10.3109/17453674.2011.552776)
Supplement: Supplementary file 1 [file ORT-1745-3674-82-090-s3843.pdf]

Supplementary data

Development of simulated arthroscopic skills

A randomized trial of virtual-reality training of 21 orthopedic surgeons

Christine Andersen[1], Trine N Winding[2], and Martin S Vesterby[2]

[1]Department of Orthopedics and [2]Orthopedic Skills Laboratory, Silkeborg Regional Hospital, Silkeborg, Denmark
Correspondence: chrisand79@gmail.com
Submitted 09-11-26. Accepted 10-08-30

Table 2. Average difference in performance from the first to the second test for the 3 groups. A positive number means an improvement from the first to the second test. Values are mean (95% CI). See Figure 4 for explanation of groups

Group	First test	Second test	Difference
Time (sec.)			
1	282 (210–355)	283 (159–407)	–1 (–99–97)
2	673 (449–896)	607 (347–868)	65 (–269–400)
3	720 (499–941)	223 (118–329)	497 (346–647)
Path camera (mm)			
1	1088 (691–1485)	1240 (603–1875)	–151 (–614–311)
2	3503 (1168–5838)	3793 (1556–6030)	–290 (2731–2151)
3	3665 (2267–5064)	836 (430–1241)	2830 (1539–4120)
Path probe (mm)			
1	1727 (774–2680)	2202 (277–4128)	–475 (–2278–1328)
2	3484 (1370–5599)	3615 (1003–6227)	–131 (–4083–3821)
3	4583 (2479–6688)	1034 (422–1647)	3549 (1717–5380)
Number of collisions			
1	175 (45–105)	96 (23–170)	–22 (–81–37)
2	145 (63–226)	175 (69–282)	–31 (–190–129)
3	197 (70–324)	68 (30–106)	129 (17–240)
Depth collisions (kN e+38)			
1	2.69 (1.76–3.63)	2.54 (1.80–3.27)	1.58 (–12.1–15.2)
2	3.13 (2.94–3.31)	3.14 (2.91–3.38	–0.18 (–3.75–3.39)
3	3.11 (2.90–3.32)	2.19 (1.44–2.94)	9.20 (1.70–16.7)

Open Access - This article is distributed under the terms of the Creative Commons Attribution Noncommercial License which permits any noncommercial use, distribution, and reproduction in any medium, provided the source is credited.
DOI 10.3109/17453674.2011.552776
